# *In silico *tandem affinity purification refines an Oct4 interaction list

**DOI:** 10.1186/scrt67

**Published:** 2011-05-13

**Authors:** Clara Yujing Cheong, Patricia Miang Lon Ng, Rhonda Ponnampalam, Heng-Hang Tsai, Guillaume Bourque, Thomas Lufkin

**Affiliations:** 1 Stem Cell and Developmental Biology Group, Genome Institute of Singapore, 60 Biopolis Street, 138672, Singapore; 2 Proteomics, Genome Institute of Singapore, 60 Biopolis Street, 138672, Singapore; 3 Computational and Mathematical Biology, Genome Institute of Singapore, 60 Biopolis Street, 138672, Singapore

## Abstract

**Introduction:**

Octamer-binding transcription factor 4 (Oct4) is a master regulator of early mammalian development. Its expression begins from the oocyte stage, becomes restricted to the inner cell mass of the blastocyst and eventually remains only in primordial germ cells. Unearthing the interactions of Oct4 would provide insight into how this transcription factor is central to cell fate and stem cell pluripotency.

**Methods:**

In the present study, affinity-tagged endogenous Oct4 cell lines were established via homologous recombination gene targeting in embryonic stem (ES) cells to express tagged Oct4. This allows tagged Oct4 to be expressed without altering the total Oct4 levels from their physiological levels.

**Results:**

Modified ES cells remained pluripotent. However, when modified ES cells were tested for their functionality, cells with a large tag failed to produce viable homozygous mice. Use of a smaller tag resulted in mice with normal development, viability and fertility. This indicated that the choice of tags can affect the performance of Oct4. Also, different tags produce a different repertoire of Oct4 interactors.

**Conclusions:**

Using a total of four different tags, we found 33 potential Oct4 interactors, of which 30 are novel. In addition to transcriptional regulation, the molecular function associated with these Oct4-associated proteins includes various other catalytic activities, suggesting that, aside from chromosome remodeling and transcriptional regulation, Oct4 function extends more widely to other essential cellular mechanisms. Our findings show that multiple purification approaches are needed to uncover a comprehensive Oct4 protein interaction network.

## Introduction

Octamer-binding transcription factor 4 (Oct4) [1], also termed Oct3 or Pou5f1 [2], is an early developmental stage transcription factor. Oct4 expression begins in the oocyte from maternal sources and is continued by zygotic expression after the four-cell stage. Thereafter it becomes restricted to the inner cell mass, the epiblast and eventually the germ cells [3]. During this time, Oct4 expression serves to regulate pluripotency and cell fate development [4]. Oct4-null mouse embryos become restricted to a trophectoderm lineage at the blastocyst stage, leading to peri-implantation lethality [5]. Such cell fate restriction is also observable in mouse embryonic stem (ES) cells when Oct4 levels decrease to less than 50% of the normal diploid expression. On the other hand, an increase in Oct4 levels by 50% converts ES cells to a primitive endodermal and mesodermal fate [6,7]. Hence, the maintenance of pluripotency requires Oct4 to be present within a very narrow concentration range, and a change in Oct4 levels directs cells to different developmental fates. Oct4 with combinations of the following factors (Klf4, c-Myc, Sox2 and Esrrb) were also shown to be sufficient to induce pluripotency in various differentiated cell types [8-10]. Therefore, Oct4 is one of the key transcription factors involved in both the maintenance of ES cell pluripotency [11,12] and somatic cell reprogramming [10,13-17]. Oct4 performs its role via switching target genes on or off. Chromatin immunoprecipitation experiments and *in silico *analyses of Oct4 have identified at least 420 target genes with putative Oct4-binding motifs [18-21]. These target genes span multiple biological processes and developmental stages. Regulation of these different genes (including *Pou5f1 *itself) has been shown to be mediated via Oct4 interaction with other transcription factors [22-24]. To better understand how Oct4 regulates a large number of genes, several studies on its protein interaction network have been attempted [25-28], and they have shown that Oct4 associates with other transcription factors and epigenetic regulators [25-28]. Here we aim to further elucidate the Oct4 interaction network using a different approach. Unlike earlier studies, our study targets the endogenous *Oct4 *allele. This approach eliminates the altering of Oct4 from its physiological levels. Although previous studies strove to keep changes in Oct4 levels within perceived limits for ES cell maintenance, it is unknown whether this minor increase in dosage would affect embryonic development. This is a very real concern, since modulating Oct4 levels is an intrinsic mechanism used by the embryo to control cell fate [6].

The two most recent studies on the Oct4 interactome [26,27] showed an overlap of about 40% of the smaller set. Is the real Oct4 interactome therefore a union or intersection of these data [29], and are these data sets sufficiently saturated to describe the Oct4 interactome? Since identical tags were used, differences between the data sets were attributed to the different preparations of ES cells. Pardo *et al. *[26] extracted total ES cell lysate in a gentle buffer using mechanical disruption, and van den Berg *et al. *[27] extracted only the nuclear extract using a high salt extraction method. Differences in data processing were another factor. These suggest that the type of interactors discovered is highly dependent on all of the experimental conditions and parameters. Therefore, the need for future studies to boost the confidence of proteins in these data sets remained [29]. Our study indicates that the Oct4 interactome can be expanded by varying purification conditions through the use of different tags to the same endogenous Oct4 protein. In all, 33 Oct4-associated proteins were identified in our study, and they associate with proteins beyond transcriptional regulatory modules. This indicates that Oct4 may utilize self-modification as a means of transcriptional regulation or may even be involved in other types of cellular processes alongside transcriptional regulation.

## Materials and methods

### Gene targeting of ES cell lines via homologous recombination

V6.4 (C57BL/6 × 129/Sv) ES cells [30] were used for gene targeting as previously described [31]. The targeting vector was constructed by inserting a floxed *neo *selection cassette (loxP-PGK-Gb2-neo-loxP) in the 5' untranslated region of the *Pou5f1 (Oct3*/*4) *gene in a mouse bacterial artificial chromosome (BAC) and insertion of a unique *Fse*I site immediately downstream of the translation start site of the *Pou5f1 *gene. Dual tags were inserted in-frame with *Pou5f1 *via the *Fse*I site. Four different constructs bearing the dual tags protein A calmodulin-binding peptide (CBP), biotin acceptor peptide (BAP)-6xHIS, S-CBP and 2xFLAG-6xHIS were made. These final constructs were used to generate the four separately tagged Oct4 ES cell lines expressing N-terminal tandem affinity purification (NTAP)-Oct4, N-terminal BAP-HIS (NBH)-Oct4, N-terminal S peptide CBP (NSC)-Oct4 and N-terminal FLAG-HIS (NFH)-Oct4. The *neo *selection cassette was removed by transient expression of Cre recombinase. For the NBH-Oct4 cell line, a second targeting vector (pROSA26-hBirA-lacZ-loxP-neo, courtesy of M. Lee) was introduced. This vector bears a "humanized" *BirA *ligase (hBirA) gene, as well as *neo *and *lacZ *markers, at the *Rosa26 *locus.

### Generation of genetically modified mice

NTAP-Oct4 and NSC-Oct4 ES cells were injected into blastocysts and used to generate tagged Oct4 chimeras that were then used to derive heterozygous and homozygous animals. The Institutional Animal Care and Use Committee at our institution approved all animal protocols used in this study.

### Affinity purification

ES cell lines for protein purification were grown without Mouse Embryo Fibroblasts (MEF). Nuclear proteins were extracted using NE-PER Reagents (Pierce Biotechnology/Thermo Scientific, Waltham, Massachusetts, USA) according to the manufacturer's instructions. Nuclear extracts were buffer-exchanged using Zeba Spin Desalting Columns (Pierce Biotechnology/Thermo Scientific) prior to purification. For S-tag purification, nuclear extract was incubated with S-protein agarose beads (Novagen, Darmstadt, Germany) in binding buffer (20 mM Tris·HCl, pH 7.5, 150 mM NaCl, 0.2% Triton X-100 and 5% glycerol), washed with four column volumes (CVs) of binding buffer and eluted with 1.4 mg/mL S peptide, KETAAAKFERQHMDS (customized peptide synthesis by Sigma, St. Louis, Missouri), in binding buffer. For FLAG-tag purification, nuclear extract was incubated with ANTI-FLAG M2 Affinity Gel (Sigma) in binding buffer (50 mM Tris·HCl, pH 7.4, and 150 mM NaCl), washed with five CVs of binding buffer and eluted with 0.1 mg/mL of 3XFLAG peptide, MDYKDHDGDYKDHDIDYKDDDDK (Sigma), in binding buffer. For HIS-tag purification, nuclear extract was incubated with Ni-NTA Superflow (Qiagen, Venlo, Netherlands) in binding buffer (20 mM Tris·HCl, pH 7.9, 350 mM NaCl, 0.2% Triton X-100 and 10 mM imidazole), washed in one CV of wash buffer, followed by two CVs of binding buffer with increased imidazole (20 mM) before elution with 300 mM imidazole in pH 7.0 binding buffer. For CBP-tag purification, nuclear extract was incubated with calmodulin-agarose (Millipore, Billerica, Massachusetts, USA) in binding buffer (10 mM Tris·HCl, pH 8.0, 150 mM NaCl, 1 mM MgOAc, 1 mM imidazole, 2 mM CaCl_2_, 0.1% Nonidet P (NP)-40 and 10 mM β-mercaptoethanol), washed in three CVs of wash buffer (50 mM Tris·HCl, pH 7.0, 350 mM NaCl, 0.2% NP-40 and 5 mM imidazole) before elution via release of the HIS-tagged calmodulin with elution buffer (50 mM Tris·HCl, pH 7.0, 350 mM NaCl, 0.1% NP-40 and 350 mM imidazole). All buffers used in protein extraction and affinity purification were supplemented with Complete Protease Inhibitor EDTA-free Cocktail (Roche, Indianapolis, IN, USA). All resins were equilibrated in binding buffer before use. The incubation time of nuclear extract with resin ranged from 1.5 hours to overnight at 4°C.

### Western blot analysis

Analyses were performed by resolving ES nuclear extraction or unbound, washed and eluted fractions from the purifications on gradient (4% to 15%) or 10% acrylamide gels by sodium dodecyl sulfate polyacrylamide gel electrophoresis (SDS-PAGE), followed by transfer of the proteins onto polyvinylidene difluoride membranes for detection with relevant antibodies. The antibodies used were anti-Oct4 antibodies (ab27985; Abcam, Boston MA, USA), anti-S antibodies (sc-802; Santa Cruz Biotechnology, Santa Cruz, CA, USA), anti-6xHIS monoclonal antibody horseradish peroxidase (HRP) conjugate (631210; Clontech, Mountain View, CA, USA), anti-CBP (sc-33000; Santa Cruz Biotechnology) and anti-FLAG M2 monoclonal antibodies (F3165 or F1804; Sigma). Unlabeled primary antibodies were detected by using HRP-linked antirabbit antibody (NA934; GE Healthcare Life Sciences, GE Healthcare Pte Ltd., Life Sciences Consumables, Singapore, Singapore), HRP-linked antimouse antibody (NXA931; GE Healthcare Life Sciences) or HRP-linked antigoat antibody (sc-2020; Santa Cruz Biotechnology). BAP tag was detected by using streptavidin-HRP (NEL750; PerkinElmer, Waltham Massachusetts, USA).

### Mass spectrometry

Proteins eluted by affinity purification were resolved by SDS-PAGE and Coomassie-stained with SimplyBlue SafeStain (Invitrogen, Carlsbad, California, USA). Each gel lane was cut into five sections. Gel pieces were subjected to in-gel tryptic digestion. Each section was submitted as an individual sample for liquid chromatography-mass spectrometry (LC-MS/MS) analysis at the Genome Institute of Singapore proteomic facility. Samples were injected using a nano-LC pump to a reversed-phase column and analyzed by using an LTQ-MS/MS spectrometer (ThermoFinnigan, San Jose, CA, USA). LC-MS/MS spectra from each purification were identified using both SEQUEST and X!Tandem search engine algorithms. The results were then loaded onto the Scaffold Proteome Software platform (Proteome Software, Inc., Portland, OR, USA), and the parameters of 95% probability of correct protein and peptide identification were set as filters.

## Results

### Endogenous tagged Oct4 cell lines express tagged Oct4 at physiological levels and remain pluripotent

**Figure 1:**
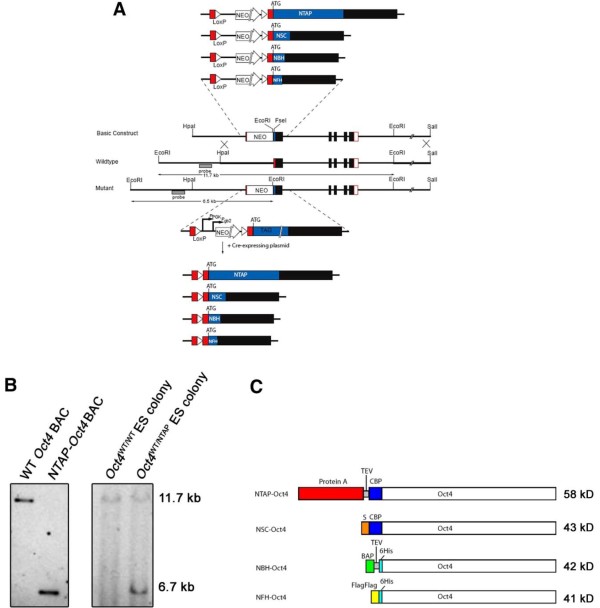
**Construction of endogenous affinity-tagged *Oct4*/*Pou5f1 *alleles by gene-targeted homologous recombination in embryonic stem cells**. **(A) **Each of four different gene-targeting constructs comprising dual-affinity tags was constructed to target the endogenous *Oct4*/*Pou5f1 *allele. NTAP (protein A calmodulin-binding peptide), NSC (S peptide calmodulin-binding peptide), NBH (biotin acceptor peptide-HIS) or NFH (FLAG-HIS) was inserted at the ATG translation start site of *Oct4*. The wild-type *Oct4 *allele gives an 11.7-kb restriction fragment during digestion with *Eco*RI, whereas correctly targeted homologous recombination results in a mutant *Oct4 *allele that gives a 6.5-kb fragment instead. These fragments were detected using an external probe that lies between the *Eco*RI site and the *Hpa*I site. **(B) **Southern blot analysis of embryonic stem (ES) cell colonies after endogenous *Oct4 *modification with the targeting construct for introduction of NTAP tag. Wild-type (WT) *Oct4 *BAC and *NTAP-Oct4 *BAC were used as positive controls for the wild-type allele fragment and the mutant allele fragment, respectively. The probe used is indicated in Figure 1A. **(C) **Diagram showing the proteins that are expressed by the respective modified *Oct4 *alleles. NBH, N-terminal biotin acceptor peptide HIS; NFH, N-terminal FLAG HIS; NSC, N-terminal S peptide calmodulin-binding peptide; NTAP, N-terminal tandem affinity purification; BAP, biotin acceptor peptide; CBP, calmodulin-binding peptide; S, S peptide; TEV, tobacco etch virus.

To look at Oct4 interacting proteins, we tagged the endogenous *Oct4*/*Pou5f1 *locus via homologous recombination (Figure 1A). Four different mouse ES cell lines were generated, each with two different affinity tags targeted to the N-terminal of Oct4. Targeting to the correct locus was verified by Southern blot analysis (Figure 1B) using a probe that lies outside the left homology arm (Figure 1A). The results show the presence of an expected shorter fragment of 6.7 kb coming from the tagged allele in addition to the 11.7-kb fragment from the unmodified allele for modified ES cells (Figure 1B). Correctly modified ES cells are annotated *Oct4*^WT/TAG^, and the proteins expressed are annotated NTAP-Oct4, NSC-Oct4, NFH-Oct4 and NBH-Oct4 (Figure 1C). The tagged Oct4 proteins that would be expressed from each of the cell lines have a span of various sizes, ranging from 41 kDa to 58 kDa, making the tagged proteins 3 kDa to 20 kDa bigger than Oct4.

**Figure 2:**
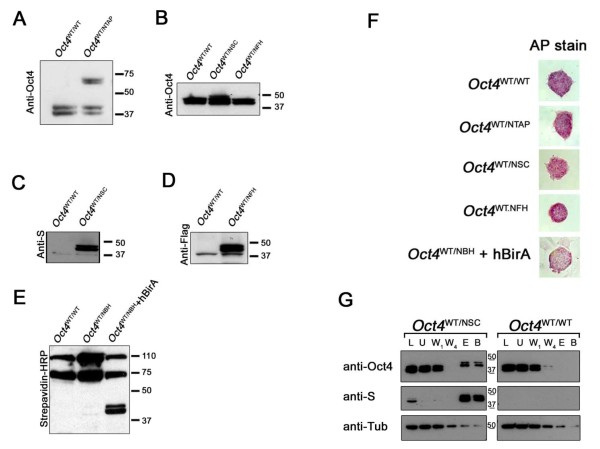
**Modified ES cells show proper expression of both wild-type and affinity-tagged alleles**. **(A and B) **Expression of NTAP-Oct4, NSC-Oct4 and NFH-Oct4 detected by anti-Oct4 shows amount of Oct4 equivalent to that found in wild-type ES cells. **(C) **Expression of NSC-Oct4 detected by anti-S antibody. **(D) **Expression of NFH-Oct4 detected by anti-FLAG antibody. **(E) **Expression of NBH-Oct4 was detected by using streptavidin-horseradish peroxidase (HRP). Wild-type ES cells were loaded as negative controls. **(F) **Alkaline phosphatase (AP) staining shows that modified ES cells are pluripotent like control *Oct4^WT/WT ^*ES cells. **(G) **S-tag affinity purification was performed on *Oct4^WT/NSC ^*ES cells and *Oct4^WT/WT ^*ES cells. Western blot analysis was performed on the nuclear lysate (L) as well as on the unbound (U) fraction, the first wash (W_1_), the last wash (W_4_), the eluate (E) and the boiled beads (B) after elution of the purification process. The marker sizes are indicated in kilodaltons. Anti-β tubulin was used as a control.

Expression levels of the various tagged Oct4 proteins from each of the four cell lines were verified by Western blot analysis (Figures 2A through 2E). Expression was not observed until the neomycin cassette, which was inserted between the endogenous *Oct4 *promoter and the translational start site of *NTAP-Oct4*, was removed. The large differences between the tagged and wild-type Oct4 sizes in the *Oct4 ^WT/NTAP ^*cell line enabled us to examine the levels of tagged Oct4 relative to wild type using anti-Oct4 antibodies. The levels are visually equivalent, confirming that the endogenously modified *Oct4 *gene retained normal levels of protein expression. It is unknown why a doublet is observed. One possibility is the posttranslational modification of Oct4, since this is also observed in the wild-type sample. For the small tagged Oct4 cell lines, loading an equal volume of total nuclear extract as wild-type ES cells produced comparable intensity when detected by anti-Oct4 antibody, suggesting that the level of Oct4 is also unaltered (Figure 2B).

Expression of the tags was confirmed by using anti-S antibody (Figure 2C) and anti-FLAG antibody (Figure 2D) for the NSC-Oct4- and NFH-Oct4-expressing cell lines, respectively. For the NBH-Oct4 expressing cell line, detection of the BAP tag was verified by using streptavidin (Figure 2E).

To evaluate the effect of each of these tags on ES cell character, we stained each of the four cell lines for alkaline phosphatase activity. All tagged cell lines stained similarly to the wild type (Figure 2F) and exhibited morphology and passage times similar to those of wild-type ES cells, suggesting that endogenous tagging does not affect ES cells.

### NTAP-Oct4 cell line produces a lethal phenotype

Although all tagged ES cell lines performed equally well in culture, the ability of tagged Oct4 to drive the formation of a whole animal was never verified. We checked for full Oct4 functionality by generating tagged Oct4 mice. Since large tags are more likely to affect protein function, we picked Oct4-tagged cell lines with the two largest tags, NTAP and NSC, for *in vivo *assessment. Mice were derived by blastocyst injection of the modified ES cells, and chimeric mice were bred to obtain heterozygous and homozygous offspring. We obtained a non-Mendelian ratio of live births for the offspring of *Oct4^WT/NTAP ^*crosses. At two weeks of age, when mice were genotyped, there was only one *Oct4^NTAP/NTAP ^*mouse (Table 1). This mouse died prior to sexual maturity. These results suggest that there was loss of function associated with the insertion of the NTAP tag at *Oct4*. Similar mating with *Oct4^WT/NSC ^*animals, however, resulted in offspring at numbers close to expected ratios. χ^2 ^testing of the data showed that the number of homozygous NSC-tagged pups obtained was consistent with the expected Mendelian ratio (Additional file 1). More importantly, *Oct4^NSC/NSC ^*homozygous mice were viable and fertile, suggesting that the insertion of the NSC tag at Oct4 did not disrupt development. Since the large NTAP tag was not tolerated in an *in vivo *system, we proceeded with affinity purifications using only three of the four original cell lines: *Oct4^WT/NSC^, Oct4^WT/NFH ^*and*Oct4^WT/NBH^*. Given the even smaller size of the NFH and NBH tags and the identical position of tag insertion in all tag variants, we postulated that these two tag variants would also would not be deleterious to Oct4 function as the NSC tag was.

**Table 1:** Number and percentage of pups belonging to each genotype as a result of mating heterozygotes^a^

	Heterozygote mating			
	***Oct4***^**WT/NTAP**^		***Oct4***^**WT/NSC**^	
Mating type	Number (%)	App	Number (%)	App
Wild-type pups (*Oct4*^WT/WT^)	25 (34.2%)	WT	41 (27.7%)	WT
Heterozygous pups (*Oct4*^WT/TAG^)	47 (64.4%)	WT	82 (55.4%)	WT
Homozygous pups (*Oct4*^TAG/TAG^)	1 (1.4%)	†	25 (16.9%)	WT
Total	73 (100%)		148 (100%)	

^a^NTAP, N-terminal tandem affinity purification; NSC, N-terminal S peptide CBP; App (appearance), longevity and fertility; WT, wild type. † = animal died

### Identification of Oct4 interacting proteins

In contrast to researchers in other studies, we identified Oct4 interacting proteins using four different affinity-tag purification approaches. The advantage of this method is that the protein interactors discovered are not limited by the conditions of one approach. Western blot analysis of the purification was used to detect the tagged Oct4 following purification from *Oct^WT/TAG ^*or from wild-type ES cells as starting material. An example of purification for the S tag (Figure 2G) shows that Oct4 is enriched only in the tagged ES cell line, but not in the wild-type ES cell line. Detection using anti-S antibody confirmed the presence of the tag in the enriched Oct4, while detection for tubulin suggested a depletion of background protein after purification amid the enrichment of NSC-Oct4. Following affinity purification, eluates were separated by gel electrophoresis. Whole lanes were excised into multiple gel bands and subjected to further tryptic digestion and peptide identification by LC-MS/MS. Raw MS/MS data were subjected to protein identification by searches using the mouse International Protein Index database (European Bioinformatics Institute, Cambridge, UK). Only proteins identified in the overlap of two separate algorithm searches (Sequest and X!Tandem) were considered confident identifications and pursued for further analysis.

**Figure 3:**
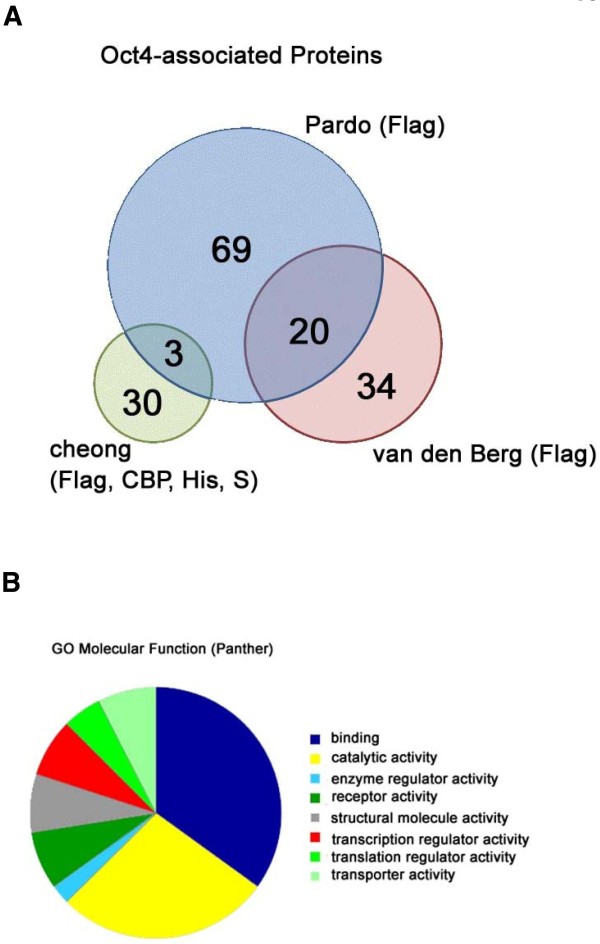
**Profile of Oct4-associated protein**. **(A) **Overlap of Oct4-associated proteins identified in three different studies. The three proteins that were shared between our Oct4 interactor lists, as well as the Oct4-associated proteins identified by Pardo *et al. *[26], were cullin 4B, importin subunit α2 and DNA topoisomerase 2α. Cheong (present study); van den Berg [27]. **(B) **Gene ontology molecular function of Oct4-associated protein according to the PANTHER database.

Although a true interactor may be found in only one of the four different approaches, we considered only proteins that were enriched by at least two approaches to keep the list robust. For better comparison with previous publications, we also used the same cutoff value: Accepting proteins are not detected in the control purifications or are threefold the control purifications or greater. On the basis of these criteria, we compiled a list comprising 33 potential Oct4 interactors (Table 2 and Additional file 2). To compare proteins identified by using different approaches with those identified by FLAG-tag purification, we overlapped the proteins in our experiment with those described in two previous studies [26,27]. Those two studies represent the most current and largest sets of Oct4 interactors identified by purification of a FLAG-tagged Oct4 transgene. Only three of our proteins (cullin 4B, importin subunit α2 and DNA topoisomerase 2α) overlapped with the proteins described in the study published by Pardo *et al. *[26], while none of our proteins overlapped with those described in the study reported by van den Berg *et al. *[27] (Figure 3A). Since the two previous studies showed 20 proteins that overlapped with each other, our proteins' degree of overlap with the proteins reported by Pardo *et al. *[26] is considerably smaller than that between studies using the same tag purifications. Consistent with this observation is that tandem purifications using two different tags resulted in only seven common proteins when results from a tandem FLAG-tag and CBP-tag purification were compared with those from a single FLAG-tag purification [26] and 30 proteins from a BAP-tag/FLAG-tag tandem affinity purification [28]. Hence, the tag used and the accompanying purification conditions change the type of interactors discovered, and the overlap is apparently small.

**Table 2:** Oct4-associated proteins using four different affinity tag approaches^a^

Gene name	Entrez Gene ID	Description	Catalytic activity based on PANTHER	CBP	FLAG	HIS	S
Pou5f1	18999	POU domain, class 5, transcription factor 1		2.0	7.0	12.0	Exc
Cell cycle associated							
Cdk1	12534	Cyclin-dependent kinase 1	Kinase	0.7	0.7	Exc	Exc
Smc2	14211	Structural maintenance of chromosome 2		0.8	Exc	Exc	
Rad50	19360	RAD50 homolog (*Saccharomyces cerevisiae*)		Exc	Exc	1.3	
Nup43	69912	Nucleoporin 43		Exc	Exc	1.2	
Metabolic processing							
Trip12	14897	Thyroid hormone receptor interactor 12		9.0	Exc	0.5	
Ribonucleoprotein complex							
Ddx1	104721	DEAD (Asp-Glu-Ala-Asp) box polypeptide 1	Helicase, translation initiation	0.5	Exc	Exc	
Dhx15	13204	DEAH (Asp-Glu-Ala-His) box polypeptide 15	Helicase	0.6	3.0	22.5	
Nop56	67134	Nucleolar protein 5A			Exc		Exc
RNA and protein transport and localization							
Kpna2	16647	Karyopherin (importin) α2		Exc	Exc		
Nup93	71805	Nucleoporin 93		1.6	4.0	Exc	
Nup85	445007	Nucleoporin 85	Transferase	3.5	Exc		
Nop58	55989	Nucleolar protein 5		1.8	Exc		Exc
Nup107	103468	Nucleoporin 107		3.3	Exc	0.8	
Rcc1	100088	Regulator of chromosome condensation 1	Ligase, small GTPase regulator, guanyl-nucleotide exchange factor	Exc	3.0		
Thoc4	21681	THO complex 4			Exc		Exc
Lbr	98386	Lamin B receptor	Oxidoreductase		Exc	3.5	
Nup153	218210	Nucleoporin 153		Exc	Exc		
Nup155	170762	Nucleoporin 155		9.5	3.6	0.9	
Tjp2	21873	Tight junction protein 2		2.0	Exc	0.5	Exc
Xpo1	103573	Exportin 1, CRM1 homolog (yeast)		3.0	Exc		
RNA processing							
Eftud2	20624	Elongation factor Tu GTP binding domain containing 2	Nucleotidyltransferase, GTPase, translation initiation and elongation	0.5	4.3	0.8	Exc
U2af2	22185	U2 small nuclear ribonucleoprotein auxiliary factor 2			Exc		Exc
Syncrip	56403	Synaptotagmin binding, cytoplasmic RNA interacting protein	RNA splicing factor, transesterification mechanism		5.0		3.0
Signal transduction							
Cul4b	72584	Cullin 4B		Exc	Exc		
Rsu1	20163	Ras suppressor protein 1		Exc		Exc	
SWI/SNF Complex							
Smarcd1	83797	SWI/SNF-related, matrix-associated, actin-dependent regulator of chromatin, subfamily d, member 1		1.0	6.0	1.0	Exc
Transcriptional regulation							
Fubp3	320267	Far upstream element (FUSE) binding protein 3		3.3	4.0		Exc
Fus	233908	Fusion, derived from t(12;16) malignant liposarcoma	RNA splicing factor, transesterification mechanism	Exc			Exc
Pelp1	75273	Proline, glutamic acid and leucine rich protein 1		Exc	Exc	0.6	
Psip1	101739	PC4 and SFRS1 interacting protein 1		2.5	Exc		4.0
Tardbp	230908	TAR DNA binding protein	RNA splicing factor, transesterification mechanism	0.6	5.0	Exc	2.0
Top2a	21973	Topoisomerase (DNA) IIα		3.7	1.8	0.7	4.0
Translational regulation							
Igf2bp1	140486	Insulin-like growth factor 2 mRNA binding protein 1	RNA splicing factor, transesterification mechanism		Exc		Exc

^a^The fold change of spectral counts of proteins from tag sample relative to wild-type sample is shown. Exc represents proteins that are seen exclusively in the tag sample.

### Oct4 interactors indicate Oct4 engagements with multiple cellular mechanisms

Of the 33 proteins, 24 were annotated by the PANTHER database [32] and are significantly enriched for proteins associated with binding activity [GO:5488] and catalytic activity [GO:3824] (Figure 3B). This suggests that proteins associated with Oct4 may determine its catalytic activity to Oct4-mediated regulation. Association with Oct4 could either lead to modifications of DNA and/or other proteins or of Oct4 itself. The exact catalytic activities associated with our proteins include transcription regulation, translation regulation, RNA splicing, helicase, kinase, ligase, GTPase, nucleotidyltranferase, oxidoreductase and transferase activities (Table 3). These activities provide a hint of the possible mechanisms that Oct4 employs. To better understand the developmental impact of these Oct4-associated proteins, we combed the Mouse Genome Informatics database for the loss-of-function phenotypes associated with the genes of these proteins. Known developmental defects are present in approximately 25% (9 of 33) of these interactors (Table 3). Aptly, all of the three transcription factors with loss-of-function phenotypes showed defects in fertility or inner cell mass formation, which is in agreement with the developmental stages in which Oct4 is known to be most essential [5,33,34]. Another six proteins not associated with transcription (Table 4) showed a range of phenotypes which could occur through either direct or indirect association with Oct4. Notably, Rad50 was a DNA repair protein that, in hypomorphic mutants, revealed a predisposition toward cancer, loss of spermatogenic stem cells and loss of hematopoietic stem cells. This suggests that Rad50 could facilitate Oct4 in its transcriptional regulatory role to control stem cell replication.

**Table 3:** Phenotypes for loss of function of Oct4-associated transcriptional regulators

Protein	Biological process	Loss-of-function phenotype
Psip1	Transcription factor activity (PANTHER)	Perinatal death (survivors show reduced fertility)
Tardbp	Transcription factor activity (PANTHER)	Embryonic lethality before somite formation with impaired inner cell mass proliferation
Fus	Positive regulation of transcription, DNA-dependent (DAVID), transcription factor activity (PANTHER)	High neonatal mortality, and male sterility associated with lack of chromosomal pairing
Top2a	Positive regulation of transcription, DNA-dependent (DAVID)	Nil
Fubp3	Positive regulation of transcription, DNA-dependent (DAVID)	Nil

**Table 4:** Phenotypes for loss of function of Oct4-associated proteins that are not known to be transcriptional regulators

Gene	Loss-of-function phenotype
Cdk1	Death prior to embryonic day 1.5
Igf2bp1	Increased neonatal lethality associated with multiple abnormalities
Lbr	Impaired growth and skin defects
Nup155	Embryonic lethality associated with atrial fibrillation
Rad50	Embryonic death. Hypomorphic mutant shows predisposition toward cancer and loss of spermatogenic and hematopoietic stem cells, leading to death.
Tjp2	Embryonic lethality associated with gastrulation defect

### Novel transcriptional regulators coenriched with Oct4

As Oct4 is a transcription factor expected to interact with other transcription factors in a modular fashion to effect transcription regulation, we were most interested in the proteins with a role in transcription regulation. Five proteins, Fubp3, Fus, Psip1, Tardbp and Top2a, were annotated by the DAVID and/or PANTHER databases as proteins with a role in transcription regulation (Table 4). Of these five proteins, Top2a has been reported to be an Oct4 interactor [26], while the other four proteins have yet to be reported.

## Discussion

Understanding the transcriptional regulatory role of Oct4 allows for the control of embryonic or induced pluripotent stem cell applications [35]. As a master regulator, Oct4 is already present in the unfertilized egg via maternal transcripts to modulate gene expression from the earliest stages of embryonic development. To coordinate gene regulation both positively and negatively in the dynamic and temporal stages of development, Oct4 presumably must interact with multiple functional modules involved in different areas of cellular regulation. Insights into such regulatory mechanisms of Oct4 can come from understanding the Oct4 protein interaction network.

Previous studies on Oct4 have employed transgenic methods that introduced a tagged Oct4 into ES cells. While care has been taken to ensure that the level of extra Oct4 does not exceed 50% of the endogenous level, an increase in Oct4 is unavoidable. Therefore, all previous work raises the concern that these changes would affect ES cells. Hence, there is a dilemma with regard to keeping the exogenous tagged Oct4 as low as possible to avoid changing cell fate and making it high so that the purification yield is better. Since our strategy does not change the endogenous level of Oct4, we can have all the Oct4 present physiologically contribute to the purification yield. Indeed, we can get detectable Oct4 by using LC-MS/MS with a low starting material level of 400 μg of nuclear extract to get a signal of 11 spectra for Oct4 in the tag purification and no signal in the wild-type control. This is a significant reduction compared to what is required (50 to 100 mg) when tagged Oct4 is expressed as a low percentage of total Oct4 [36,37]. Also, since the endogenous Oct4 is modified, the presence of untagged Oct4 acting as a competitor for interactors is reduced.

While keeping Oct4 to its endogenous level is important, no previous study has addressed a separate concern that the tags used may impede the function of Oct4. We tested two different tags in animals and found that the classically used NTAP tag [38,39], comprising two protein A and one calmodulin-binding protein, prevents Oct4 from driving embryonic development normally. This information is especially useful for future work involving gene tagging in both *in vitro *and *in vivo *studies.

Three of four previous reports on Oct4 protein interaction network used the FLAG tag in their approach. The protein interactors found showed overlaps, lending confidence to what are identified as true Oct4 interactors. However, the use of a similar tag means that common contaminants raised from a specific affinity purification will also be repeatedly identified. Although tandem affinity tags have previously been used [26,28], the number of proteins found was lower than when single purifications are used, suggesting that the inclusion of different tags can produce a low overlap. One problem with tandem affinity purification is the loss of yield as the number of steps and experimentation time increase. To overcome this, we simply performed the purifications using different tags on fresh ES cell samples and performed *in silico *tandem affinity purification instead. This method allowed us to discover a total of 33 proteins using a low amount of starting material (400 μg of nuclear extract) per purification.

In the online discussion by Pardo *et al. *[26] following his publications on Oct4 interactors, this study group suggested that heterogeneity in data sets can arise from the cell line, the tagging strategy and particularly the purification procedure used. Therefore, to expand the list of interactors from the previous data sets, we tested whether we could find novel interactors that bind to Oct4 under different purification procedures using different tags from different cell lines. Although our approach for protein extraction is similar to that of van den Berg *et al.*, our purification procedure following protein extraction is different from those used in studies by both van den Berg *et al. *[27] and Pardo *et al. *[26]. Additionally, we included a different combination of tags for analysis. Using purification buffers for each affinity tag that differ by parameters including ionic charge, pH and the use of detergents, we intended to identify a group of Oct4 interactors that were robustly identified across varying conditions. These would be representative of stalwart Oct4 interactions that could occur despite the microenvironments that might arise in a cell. Also, by considering only interactors that remained bound to Oct4 under at least two different purification procedures, we ensured that these interactions were not an artefact of a specific purification procedure alone.

The focus of Oct4 interaction has been on chromatin modifiers and transcriptional factors. With the use of different approaches of purification, the majority of our proteins showed other catalytic activities in addition to transcriptional regulation. These include helicases, ligases and RNA processing. Oct4 may recruit these enzymes to modify itself or its associated proteins as an additional means of regulation. Consistent with this hypothesis is a report that Oct4 associates with the glycosylating enzyme Ogt [27], suggesting that posttranslational and splicing activities should not be overlooked in considering Oct4 function. Indeed, Oct4 and other transcription factors have been shown to regulate their activity via *O*-GlcNAc modification [40-42]. Sumoylation of Oct4 has also been reported to enhance its stability, DNA binding and transactivation [43]. In a separate study, Oct4 was reported to be ubiquitinated by Wwp2, an E3 ubiquitin ligase [44]. Beyond modification of Oct4 activity levels, association with enzymes could give Oct4 the ability to modify DNA or other proteins. Potentially, Oct4-associated helicases could be recruited to Oct4-mediated transcriptional sites to keep the genome stable. In view of the fact that ES cells and embryos are both systems that require rapid DNA replication and transcription [45], there is a need for helicases to keep the genome stable when replication and transcriptional complexes collide [46].

In addition to transcription-related activities, Oct4 also affiliates with proteins involved in cell cycle regulation. Cdk1 has previously been established in an Oct4 interaction network [28] and is critical for the self-renewal of ES cells [47]. Because of the unique cell cycle phasing of ES cells with a short G_1_/S phase that promotes rapid proliferation [48-50], the coupling of Oct4 activity with cell cycle kinases such as Cdk1 may be necessary for rapid and direct coordination of genomic activity with cell division, failing which apoptosis may occur, as shown by ES cells depleted of Cdk1 [47]. A separate Oct4 interactor discovered in our study, Smc2, forms part of the condensins I and II protein complexes required for proper DNA compaction during the interphase [51,52]. An RNA interference screen in ES cells identified Smc2 as a protein essential for proper chromosomal compaction in ES cells, and a deficiency in Smc2 results in metaphase arrest in these cells [53]. As ES cells are known to maintain much of their chromatin in a heterochromatin state [54], there is a significant role for Oct4 in mediating both epigenetic machinery and condensin complexes to enable the removal of activating histone modifications that can perturb proper compaction for mitosis.

Cul4b has also previously been identified as an Oct4 interactor [26] and is an E3 ubiquitin ligase [55]. Interestingly, like Oct4, Cul4b is involved in Wnt signaling through its repression of nuclear β-catenin levels that can otherwise serve as a positive factor for differentiation [56]. Separately, Oct4 and Cul4b have also been shown to interact with β-catenin in immunoprecipitated complexes [56-58].

In addition to the role of Oct4 in cell cycle regulation and inhibition of differentiation through the Wnt pathway, it appears that Oct4 also associates with a number of proteins involved in nuclear transport. While Kpna2 is a previously known Oct4 interactor [59] itself, Kpna2 and another novel Oct4 interactor, Rcc1, are both specifically involved in the nuclear import of proteins [60]. In our study, various nucleoporins were also found to associate with Oct4, which suggests that a complex comprising nuclear pore proteins supporting factors such as Kpna2 and Rcc1 work in tandem with Oct4, although their imported cargo is as yet unclear. Given the known link between Rcc1 and chromatin [61], it is highly plausible that Oct4 utilizes Rcc1 as an intermediary between the current chromatin state and the transport of necessary proteins for gene expression from their cytosolic compartment.

The diversity in function of novel and known Oct4 interactors identified in our work clearly highlights the need for a multifaceted approach for the completion of the Oct4 interaction network. We believe that our use of endogenous tagging methods and a combined *in silico *analysis of identified proteins from different purification conditions serves as a resource for research in this direction. While future work should include validation of the interactors and a detailed investigation into the molecular mechanism of each interactor, we are confident that our present findings are of great value in expanding our framework for understanding Oct4 interactions beyond transcriptional control alone.

## Conclusions

In summary, we have used endogenously tagged Oct4 to study its interaction under physiological Oct4 levels. The use of multitag purification platforms allowed for a wide scope in the discovery of interactors. The proteins identified in this study include novel transcription factor interactions and a demonstrated role for Oct4 in ES cells that involves catalytic activities other than transcriptional regulation.

## Abbreviations

CV: column volume; ES: embryonic stem; NBH: N-terminal biotin acceptor peptide HIS; NFH: N-terminal FLAG HIS; NSC: N-terminal S peptide calmodulin-binding peptide; NTAP: N-terminal tandem affinity purification.

## Competing interests

The authors declare that they have no competing interests.

## Authors' contributions

CYC participated in the design of the study, carried out the experimentation, helped in the interpretation of the results and helped to draft the manuscript. PMLN carried out the experimentation, helped in the interpretation of the results and helped to draft the manuscript. RP and HHT carried out the experimentation. GB participated in the interpretation of the data. TL conceived of the study, participated in its design and coordination and helped to draft the manuscript. All authors read and approved the final manuscript.

## Supplementary Material

Additional file 1 **Supplemental Table S1**. χ^2 ^testing of pups from heterozygote mating. Click here for file

Additional file 2 **Supplemental Table S2**. Spectral counts of Oct4-associated proteins identified using four different affinity tag approaches. Click here for file
